# Evaluation of *Pachycrepoideus vindemiae* and *Muscidifurax raptor* (Hymenoptera: Pteromalidae) as biological control agents of *Piophila casei* (Diptera: Piophilidae) in ham production facilities

**DOI:** 10.1093/jisesa/iead067

**Published:** 2023-09-18

**Authors:** Diletta Missere, Antonio Martini, Giovanni Burgio

**Affiliations:** Department of Agricultural and Food Sciences (DISTAL), Alma Mater Studiorum Università di Bologna, Viale Fanin 42, 40127 Bologna, Italy; Department of Agricultural and Food Sciences (DISTAL), Alma Mater Studiorum Università di Bologna, Viale Fanin 42, 40127 Bologna, Italy; Department of Agricultural and Food Sciences (DISTAL), Alma Mater Studiorum Università di Bologna, Viale Fanin 42, 40127 Bologna, Italy

**Keywords:** Pteromalidae, Piophilidae, stored product, biological control, parasitoid

## Abstract

Ham products play a fundamental role in the Italian economy, and attention to the problems of this sector is essential. The products of this sector can be attacked by parasites, which can cause direct and indirect damage. *Piophila casei* (L.) (Diptera: Piophilidae) a cheese and meat parasite, is currently responsible for hygiene problems in ham factories. The trophic activity of this pest on the products causes serious direct damage and it is a vector of various bacteria, including *Clostridium botulinum*. Another risk is human ingestion of the larvae, which are resistant to gastric juices action, potentially causing intestinal myiasis. Insecticide use of any type is not allowed in aging rooms, so biological control can represent a potential alternative. In this study, we investigate quality parameters such as successful rate of parasitism (SP), degree of parasitism (DP), sex-ratio (SR), life-span (LS), and emergence rates (ER) of 2 pupal parasitoids of Diptera: *Pachycrepoideus vindemiae* (Rondani) (Hymenoptera: Pteromalidae), currently the only known pupal parasitoid of *P. casei*, and *Muscidifurax raptor* (Girault and Sanders) (Hymenoptera: Pteromalidae). Our research confirmed *P. vindemiae* efficacy to parasitize *P. casei* and reported, for the first time, *M. raptor* as a pupal parasitoid of this Piophilidae. ER for both parasitoids were low, thus affecting the DP and SP estimations. This could be explained by the feeding behavior of the parasitoid host. The strongly female-biased SR for *P. vindemiae* supported previous studies. LS results in our experiment are crucial for determining the timing of release.

## Introduction

The ham production sector in Italy is a fast-growing industry with a net worth of about €8.8 million ($9.4 million USD) in 2021. Dry-cured ham is the main cured meat product with a value of €2.3 million ($2.5 million USD; ASSICA 2022). Production of cured meats takes place in environments characterized by constant thermo-hygrometric conditions that are close to the optimum for many arthropod pests. Addressing arthropod pest problems associated with the production of cured meats is essential to maintaining this industry. For example, the curing of hams takes place in temperature-controlled rooms between 14 and 25 °C, a favorable microclimate for arthropods like ham mites, red-legged beetles, flies, and larder beetles (Zhao et al. 2016).

*Piophila casei*, a cosmopolitan fly is one of the most frequent and economically important pests in ham production. This pest is an excellent invader of processed food industries due to its characteristics: tolerance to a wide range of temperatures, many generations per year, and a high fertility rate. Once this fly invades a facility, the larvae can rapidly spread through the food products because of its ability to “jump” from one product to another. Larval skipping is accomplished by the larva grasping small protrusions on the anal segments with its mouth hooks, curling into a C-shape, and then suddenly releasing the mouth parts. This behavior is why the common name for *P. casei* is “the cheese skipper”. The adult flies lay their eggs near the top of the femur or in cracks that form in hams as they are hung to cure. When the larva is fully developed, it leaves the food, searching for suitable pupation sites like on the floor (Derat Parma et al. 2020).

The larvae cause damage either directly by feeding on the curing ham, or indirectly by contaminating the ham. The larvae directly feed on the exposed soft tissue, entering either around the inner bone or near the shank end where the rope used for hanging is located (Derat Parma et al. 2020, Arboix 2021). The larval feeding activity contaminates the ham products and may cause gastric and intestinal myiasis in humans that unintentionally ingest larvae contaminating the ham (Nocera and Crotti 2009). The adult flies may also vector serious human pathogens, such as Listeria monocytogenes and Clostridium botulinum, because of the strong attraction to proteins in various stage of decomposition (Domenichini 1991, 1997, Lewis and Kaufman 2010). The presence of this insect in ham products clearly affects marketability and food safety.

Current management of *P. casei* in cured meat production relies on exclusion, prophylactic, and sanitation tactics used together. Sanitation tactics include regular thorough cleaning of all production areas, equipment, and utensils and/or fumigation or insecticide applications with insecticides with residual action (Derat Parma et al. 2020). Recent restrictions on the use of insecticides and fumigants in food production areas and problems with insecticide resistance in populations of *P. casei* are negatively impacting current management programs (Rossi and Presciuttini 1996, Fields and White 2002). Clearly, additional management tactics are needed. Killing *P. casei* in storage rooms is difficult due to the deep nature of the infestations and the need to avoid undesirable changes in the treated meat.

Biological control may be useful to incorporate into the current management program because this tactic may provide a safe, sustainable method of *P. casei* management without the issues brought on by reliance on insecticides (Rossi and Presciuttini 1996, Fields and White 2002, Schöller 2010, Russo 2011). Concerns have been raised about potential food contamination by arthropods (e.g., biological control agents), regulations that prohibit the sale of contaminated food products, and negative consumer and processor perceptions (Hervet and Morrison 2021). However, studies conducted in the management of other stored product arthropod pests have shown that use of parasitoids to control the pest did not result in an increase in the arthropod fragments in the stored products (Flinn and Hagstrum 2001). Any remaining arthropod fragments could be removed from the stored products using standard cleaning procedures (Hervet and Morrison 2021).

In this study, we investigated the use of 2 pupal parasitoids to manage populations of *P. casei* in ham production facilities because this insect pupates away from the ham products, thereby avoiding problems of food contamination. The 2 parasitoids were *Pachycrepoides vindemiae* (Rondani) and *Muscidifurax raptor (*Girault and Sanders) (Hymenoptera:Pteromalidae). Both natural enemies are ectoparasitic idiobiont parasitoids (Godfray 1994, Tucker and Kaufman 2017) and attack pupae of many dipteran species (Wang and Messing 2004, Tucker and Kaufman 2017, Biancheri et al. 2022). The specific aim of this study was to quantify the quality parameters: successful rate of parasitism (SP), degree of parasitism (DP), sex-ratio (SR), and life-span (LS) for each parasitoid (Prevost 2009). Investigating these parameters is the first step in evaluation of their potential efficacy against *P. casei*. Additionally, these parameters assist in designing mass rearing procedures and provide baseline data for determining release rates and timing of releases.

## Materials and Methods

### Insect Origin and Rearing

Rearing *P. vindemiae*, *M. raptor*, *P. casei*, and *Drosophila suzukii* was conducted in the entomological laboratory of the Department of Agricultural and Food Sciences (DISTAL) of the University of Bologna. All insect colonies were maintained at 25 °C ±1 °C, 50–60% RH, photoperiod 16L:8B in a walk-in climatic chamber. The rearing program started in the summer of 2020. Field-collected individuals were introduced into the colony every month to avoid inbreeding and genetic drift caused by the maintenance of relatively small populations in captive-rearing conditions for a long period (Woodworth et al. 2002, Stouthamer 2017). *Musca domestica*, used as a secondary host to avoid habituation of a parasitoid to *P. casei*, was supplied as pupae from Bioecology, S.R.L. (Via della Corte, 4, 42025 Corte, Tegge RE).

### *P. casei* Rearing

The colony of *P. casei* was established from wild specimens collected in a cured meat factory located in Traversetolo (PR) (NE Italy). Adult flies were kept in 5 Plexiglas cages (13 × 36 × 24 cm) provided on each side with an opening covered with plastic mesh for ventilation. The flies were fed with an artificial diet consisting of 50 g L^−1^ dead yeast, 20 g L^−1^ agar, 80 g L^−1^ powdered milk, 1/10 g mlnipagine/alcohol, and via cotton balls soaked in a sugar and water solution (20% sugar). Fresh diet was provided every 2 days, inserting 5 cylinders (height 6 cm, diameter 3 cm) in each bug dorms, each containing 15 ml of artificial diet and used as oviposition substrate and moisture source. After the oviposition, the cylinders were closed with perforated lids and transferred to a refrigerator. Sterile gauze compresses are used to support the cotton wool inserted inside the cylinder in order to let the larvae pupate. For this step, it is necessary to use a mesh size gauze to facilitate the larvae’s passage. Consequently, the pupae are easily picked by stripping the cotton containing the larvae (Sacchi et al. 1971).

### *D. suzukii* Rearing

The colony was initiated from field collections on wild *Rubus* spp., in the garden of our department. Similarly to Mazzetto et al. (2016), adult flies were kept in Plexiglas cages (20 × 20 × 20 cm) and fed via cotton balls soaked in a honey and water solution (20% honey). Twice a week, 3 cylinders (height 6 cm, diameter 3 cm) containing 15 ml of the diet (1,600 ml water, 50 g sucrose, 150 g maize flour, 50 g dead yeast, 4/16 g ml^−1^ nipagine/alcohol, and 10 g agar) were inserted in the cage and maintained for 2–3 days to obtain oviposition. The cylinders removed were closed with lids and transferred to a growth chamber for larvae rearing.

### *P. vindemiae* and *M. raptor* Rearing

**Fig. 1.**
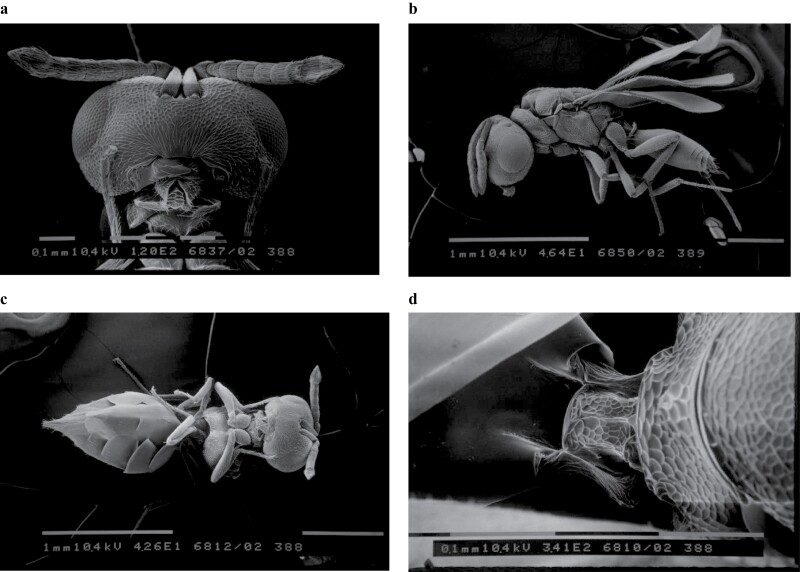
*Pachycrepoideus vindemiae* SEM image: a) head; b) profile view; c) gastro; d) petiole.

The rearing of *P. vindemiae*, was started by a population collected in the garden of our department, using Petri dishes containing pupae of *D. suzukii*. The identification to species level of *P. vindemiae* was carried out using the dichotomous keys proposed by Bouček and Rasplus (1991), relating to the main characters of the genera of the family Pteromalidae. High-resolution photographs of the morphological characters for identification were taken, using a scanning electron microscope (Philips 515) (Fig. 1). The samples were preserved in 95% ethanol, then transferred to absolute ethanol (100%) for 24 h, in chloroform for 30 min, and finally mounted on a stub to be metalized with gold and observed at 15 kW. The colony of *M. raptor* was started from adult wasps emerging from parasitized pupae of *M. domestica* provided by Bioecology S.R.L. The emerged parasitoid adults of both species were kept inside Plexiglas cages (20 × 20 × 30 cm) closed at the top by a fine mesh net (<1 mm^2^ mesh size) and fed with honey drops placed on small pieces of paper and a dispenser of water and sugar (20% sugar). Petri dishes containing ad libitum *P. casei* pupae ≤3 days old were provided every 2 days and the removed plates were kept in environmental chamber.

### Standard Protocol Used in Lab Experiments

Tests were conducted on the first and second generations, in order to assess the performance trend in subsequent generations. All the tests were conducted at 25 °C ± 1 °C, 50–60% RH and 16 L:8 D photoperiod. The tests were carried out using *P. casei* 2- to 3-day-old puparium to avoid the prepupal stage which when attacked can result in the death of the parasite progeny (Wang and Messing 2004). For all the tests, we used *P. vindemiae* and *M. raptor* ≤3 days old females. The parasitoid females were kept with males for 24 h from adult emergence. In fact, the females of *M. raptor* and *P. vindemiae* are immediately receptive to mating upon emergence from the host puparium (Crandell 1939, Tucker and Kaufman 2017). The parasitoids were provided with honey and water, because water-deprivation could increase host-feeding (Bezerra Da Silva et al. 2019a).

### Successful Rate of Parasitism, Degree of Parasitism, Emergence Rate (ER): No-choice Test

To evaluate the parasitism ability of *P. vindemiae* and *M. raptor* on *P. casei*, no-choice tests were carried out (Mazzetto et al. 2016). In no-choice tests, 10 pupae of *P. casei* were put into a plastic dish (height 2 cm, diameter 5.5 cm) with a female of the parasitoids. After 24 h, the female was removed, and each dish was checked daily to detect parasitoid or fly adult emergence. For each parasitoid species, at least 10 replicates (depending upon generation) were performed each consisting of 10 pupae; the same number of replicates with 10 *P. casei* pupae, without parasitoid, were used as a control, to check the fly emergence. The number of successfully parasitized pupae (i.e., pupae from which a parasitoid adult emerged), and number of dead pupae (i.e., pupae from which neither a parasitoid nor a *P*. *casei* adult emerged) were evaluated.

### SR

The wasps used in no-choice test were not exposed to other female wasps because the scent of another female could increase the offspring sex-ratio produced (Koul and Dhaliwal 2003). The pupae used in the precedent test were of similar size, because females tend to deposit male offspring in smaller hosts and female offspring in larger hosts (Prevost 2009). Newly emerged adult parasitoids from the no-choice test were collected daily, counted, and sexed.

### LS

LS of the parasitoids was divided into 2 phases: the development time (DT) between egg hatching and adult eclosion, and the period of adult life, referred to as longevity (LO) (Blackburn 1991). Therefore, newly eclosed adults of *P. vindemiae* and *M. raptor* emerged from the no-choice test, were individually placed in a plastic vial (height 10 cm, diameter 2 cm) closed with a mesh cap. The wasps were fed daily with honey drops mixed with water applied to the cap mesh. The date of parasite window, the length of time for which the pupae host is available to the parasitoids (Blackburn 1991), and the date of emergence respective to each parasitoid were recorded to figure out the DT. The vials with the wasps were checked every day to record the death date needed to determine longevity.

### Data Analysis

Statistical analyses were performed using IBM SPSS Statistics (IBM Corp. 2019) (ver. 26). We used 2 indices that summarize the host–parasitoid interactions (described by Biondi et al. 2021).

To estimate the impact of host species on the development of parasitoid offspring, SP was used. It provides the probability that a parasitized host would give rise to an adult wasp and it is estimated as: ep/(ef − efp), where ep = number of emerged parasitoids, ef = the average number of emerged flies in the absence of parasitoids (control), and efp = number of emerged flies in the presence of parasitoids. In instances when ep > (ef − efp), we set SP = 1. The DP calculates the proportion of hosts that were successfully parasitized. It is similar to the Abbott or Schneider-Orelli formula, which is used to correct for treatment-related mortality, taking into account control influence. It is calculated as: (ef − efp)/ef, when ef < efp, we set ef – efp = 0. We calculated the “emergence rate” (ER) as the ratio of the number of emerged parasitoids to the total number of pupae, without taking into account the parasitoids mortality.

Differences in DP, SP, ER, DT, LO recovered from the first and second generation of *P. vindemiae* and *M. raptor*, were analyzed by the nonparametric Mann–Whitney *U*-test (*P* < 0.05). A chi-square statistic was used to test for potential changing of the sex-ratio between the first and second generation progeny.

## Results

### P. vindemiae

We observed a high DP by *P. vindemiae*, reaching a value of 75.41 ± 10.74 in the first generation and 74.10 ± 6.06 in the second (Table 1). Estimated DPs did not show any statistically significant differences between the 2 generations by Mann–Whitney test (*U* = 94.00; *P* = 0.812). By pooling data from no choice tests, the SP (%) was 41.44 ± 10.30 in the first generation; the second generation showed a significantly lower SP (%), 22.01 ± 5.54 (Table 1). In this case, SP proved to be significantly different between the 2 generations (*U* = 51.50; *P* = 0.031). ER (%) of *P. vindemiae* were 26.00 ± 4.73 and 12.00 ± 3.44 in first and second generation, respectively, with a significant difference (*U* = 50.00; *P* = 0.031; Table 1).

**Table 1.** Parameters (mean ± SE) of *Pachycrepoideus vindemiae* investigated in the first generation and in the second one. DP = Degree of parasitism; SP = Successful rate of parasitism; ER = Emergence rate; DT = Development time; LO = Longevity; SR = Sex-ratio. Differences in the measures covered from first and second generation, were analyzed by the nonparametric Mann–Whitney *U*-test (*P* < 0.05). Differences of sex-ratio between the 2 generation offsprings was statistical analyzed using chi-square test

*Pachycrepoideus vindemiae*	First generation	Second generation	Mann–Whitney *U*		Sig.
DP (%)	75.41 ± 10.74	74.10 ± 6.06	94.00		0.812
SP (%)	41.44 ± 10.30	22.01 ± 5.54	51.50		0.031
ER (%)	26.00 ± 4.73	12.00 ± 3.44	50.00		0.031
DT (days)	25.42 ± 0.77	21.12 ± 0.35	99.00		*P* < 0.001
LO (days)	21.30 ± 1.97	29.58 ± 1.45	473.0		0.002
	**First generation**	**Second generation**	**Chi-square test**	**Degrees of freedom**	**Sig.**
**Sex-ratio (%)**	76.92	87.5	0.95	1	0.331

The LS of *P. vindemiae* in the first generation was 46.73 ± 2.09 days, with a development time of 25.42 ± 0.77 days and a longevity of 21.30 ± 1.97 days. The LS of the parasitoid in the second generation was 50.71 ± 1.34 days. *Pachycrepoideus vindemiae* showed a significantly (*U* = 99.00; *P* < 0.001) faster DT in *P*. *casei* (21.12 ± 0.35 days) in the second generation compared to the first (Table 1). Also, in the first generation, the LO was significantly higher than that of the second (*U* = 473.0; *P* = 0.002), reaching a value of 29.58 ± 1.45 days (Table 1). SR was 76.92% (female:male) (20 females and 6 males) in the first generation and 87.5% in the second (21 females and 3 males). Statistical analysis detected no significant differences of SR between the 2 generation offsprings (chi-square test = 0.95; df = 1; *P* = 0.331; Table 1).

### M. raptor

In the no-choice, *M. raptor* females accepted *P. casei* pupae and its progeny was able to complete development. DP (%) was 50.44 ± 8.68 in the first generation; a higher DP was detected in the second generation of *M. raptor* (75.78 ± 5.85) (Table 2), resulting a difference very close to the significance level (*U* = 76.00; *P* = 0.052). The SP (%) was 30.00 ± 9.00 and 23.54 ± 4.91, in the first and second generations, respectively (*U* = 46.50; *P* = 0.796; Table 2). ER (%) of *M. raptor* were 13.00 ± 2.47 and 19.00 ± 4.11 in first and second generations, respectively (*U* = 64.00; *P* = 0.315; Table 2).

**Table 2.** Parameters (mean ± SE) of *Muscidifurax raptor* investigated in the first generation and in the second one. DP = Degree of parasitism; SP = Successful rate of parasitism; ER = Emergence rate; DT = Development time; LO = Longevity; SR = Sex-ratio. Differences in the measures covered from first and second generation, were analyzed by the nonparametric Mann–Whitney *U*-test (*P* < 0.05). Differences of sex-ratio between the 2 generation offsprings was statistical analyzed using chi-square test

*Muscidifurax raptor*	First generation	Second generation	Mann–Whitney *U*		Sig.
DP (%)	50.44 ± 8.68	75.78 ± 5.85	76.00		0.052
SP (%)	30.00 ± 9.00	23.54 ± 4.91	46.50		0.796
ER (%)	13.00 ± 2.47	19.00 ± 4.11	64.00		0.315
DT (days)	19.00 ± 0.35	21.96 ± 0.24	1,061		*P* < 0.001
LO (days)	22.00 ± 1.74	29.05 ± 1.36	879.50		0.001
	**First generation**	**Second generation**	**Chi-square test**	**Degrees of freedom**	**Sig.**
**SR (%)**	43.47	61.53	2.11	1	0.146

The LS of *M. raptor* in the first generation was 40.00 ± 1.14 days, with a DT of 19.00 ± 0.35 days, and a longevity of 21.96 ± 0.24 days. In the second generation of *M. raptor*, the LS was 50.73 ± 1.39 days, with a development time of 22.00 ± 1.74 days, and 29.05 ± 1.36 days of longevity. DT showed a significant difference between the 2 generations (*U* = 1061; *P* < 0.001; Table 2). Also, the longevity was significantly longer in the second generation compared to the first generation (*U* = 879.5; *P* = 0.001; Table 2). The SR was 43.47% and 61.53% (females:males) (10:13 and 32:20) in the first and second generations, respectively (χ^2^ test = 2.11, df = 1, *P* = 0.146; Table 2).

## Discussion

Biological control using parasitoids may be a sustainable method to respond to the urgent need to manage this pest. To aid in incorporating biological control into a management program, quality parameters associated with each parasitoid must be determined. To aid in incorporating biological control into a management program, quality parameters associated with each parasitoid must be determined. Quantifying these parameters assist in designing mass rearing protocols and in developing release programs (Morales-Ramos et al. 2022).

The analysis of DP, SP, ER, DT, LO, and SR in this study suggested that both parasitoid species could successfully parasitize pupae of *P. casei*. This study is also the first to report that *P. casei* can be used as a host for *M. raptor*. The estimated SP and DP parameters also suggested that both parasitoids could decrease densities of *P. casei*. However, the low ER of the parasitoids from the pupae suggests that SP and DP may be overestimated. This may be due to either host feeding by the adult parasitoid or a physiological incompatibility between parasitoid and host (Chabert et al. 2012). Both parasitoids are known to host feed, resulting in the death of the host and immature parasitoid (Tucker and Kaufman 2017, Bezerra Da Silva et al. 2019b). Further studies are needed to determine the amount and impact of host feeding and what, if any, physiological incompatibility exists between *P. casei* and the 2 parasitoids. Our results demonstrated a strongly female-biased SR for the parasitoid *P. vindemiae*, corroborating previous studies (Nøstvik 1954, Nadel and Luck 1985). The sex-ratio of the progeny was determined in this study because it is used to measure the quality of biological control agents produced in mass-rearing programs (Morales-Ramos et al. 2022). In addition, the SR of the biological control agent must be considered when determining which release method (i.e., inoculative versus inundative) to use in a management program (Koul and Dhaliwal 2003). SR of the biological control agents released can be a key factor in the success of the biological control program. The strong differences in quality parameters between the first and second generation parasitoid progeny found in this study must be considered when designing both rearing protocols and field release tactics. The ER for both parasitoids were low in both generations possibly due to host feeding or physiological incompatibility. Therefore, the other quality parameters such as DP, SP, SR, and LS must be considered before implementing a biological control program. The DP and LS found in this study suggest the potential for either parasitoid to reduce densities of *P. casei* if released using an appropriate strategy. However, more research is needed under field conditions. Estimating quality parameters for parasitoids in the laboratory prior to use in a biological control program is a critical first step to predict potential impact on a target pest population and planning for mass rearing of the parasitoid (Cerutti and Bigler 1995, Morales-Ramos et al. 2022).
